# A novel computed tomography radiomic nomogram for early evaluation of small airway dysfunction development

**DOI:** 10.3389/fmed.2022.944294

**Published:** 2022-09-13

**Authors:** Sijia Cui, Zhenyu Shu, Yanqing Ma, Yi Lin, Haochu Wang, Hanbo Cao, Jing Liu, Xiangyang Gong

**Affiliations:** ^1^Rehabilitation Medicine Center, Department of Radiology, Zhejiang Provincial People’s Hospital, Affiliated People’s Hospital, Hangzhou Medical College, Hangzhou, Zhejiang, China; ^2^Hangzhou Medical College, Institute of Artificial Intelligence and Remote Imaging, Hangzhou, China

**Keywords:** small airway dysfunction, risk factor, pulmonary function test, radiomics, nomogram

## Abstract

The common respiratory abnormality, small airway dysfunction (fSAD), is easily neglected. Its prognostic factors, prevalence, and risk factors are unclear. This study aimed to explore the early detection of fSAD using radiomic analysis of computed tomography (CT) images to predict fSAD progress. The patients were divided into fSAD and non-fSAD groups and divided randomly into a training group (*n* = 190) and a validation group (*n* = 82) at a 7:3 ratio. Lung kit software was used for automatic delineation of regions of interest (ROI) on chest CT images. The most valuable imaging features were selected and a radiomic score was established for risk assessment. Multivariate logistic regression analysis showed that age, radiomic score, smoking, and history of asthma were significant predictors of fSAD (*P* < 0.05). Results suggested that the radiomic nomogram model provides clinicians with useful data and could represent a reliable reference to form fSAD clinical treatment strategies.

## Introduction

Small airway refers to the bronchioles located at the end of the bronchus, whose luminal diameter is < 2 mm. Most bronchioles are terminal bronchioles, which are important for lung gas exchange (1, 2). Initially, small airway blockage results in little detectable change in airway resistance, producing a small airway known as “the silent zone of lung disease” (3). Unfortunately, as the disease progresses, small airway obstruction detrimentally affects lung function (4, 5). Almost 40% of Chinese adults have small airway dysfunction (fSAD) defined by lung function tests (6). Therefore, maintaining the normal physiological function of small airways is significant for respiratory health, and the early detection of small airway lesions helps to inhibit respiratory disease progression.

Inflammation or stenosis of the small airway are the main causes if fSAD and small airways abnormalities are characterized by premature air trapping and airway closure,

and the increased limitation of the dependence on airflow volume (7). Without treatment or improvement, the body enters a state of long-term oxygen insufficiency. Recent research demonstrated that in patients with chronic obstructive pulmonary disease (COPD), the characteristics of the small airways are peribronchial fibrosis, hyperplasia of airway smooth muscle, inflammatory cell infiltration of the airway wall, and thickening of the airway wall with epithelial changes. Pathological evidence of emphysema is preceded by a marked loss of small airways (8). Furthermore, in asthma, chronic epithelial inflammation thickens the small airways, correlating with asthmatic exacerbation and the increased severity/frequency of dyspnea (9). This was supported by the observation that fSAD occurs prior to CT detection of emphysema and spirometric evidence of COPD, and is thus considered a precursor of asthma and COPD (10).

To assess whether early or high-risk groups have lung diseases, small airway function can be measured. However, detecting early physiological abnormalities in small airways is challenging. In addition to the spirometry test, high resolution CT (HRCT) scanning can be used to detect small airway injury at the early stage; however, it needs? to detect the degree of air retention by dual phase breathing (11). Certain quantitative imaging features can reveal the shape of the lung, the intensity changes, and fine and rough lung tissue structures. For example the long run high gray level emphasis (LRHGLE) reflects the image intensity and uniformity, and quantifies the relationship between image attenuation and tissue uniformity (12).

However, there is no research proving the relationship between early lung imaging findings and the late occurrence of fSAD in patients. Our research aimed to explore whether early changes in lung CT microstructure are predictive of fSAD, to evaluate the factors influencing fSAD, leading to early fSAD prediction by non-invasive methods, and to understand its risk factors (i.e., the associations among fSAD, lifestyle, and environment), allowing early clinical intervention delayed disease progression.

In this study, we used radiomic feature to assess the risk of fSAD, and together with the with radiomic score (rad-score) and clinical risk factors, established a predictive model (radiomic nomogram). This model might aid precise treatment and clinical decision-making of fSAD in the future.

## Materials and methods

### Ethical approval

The Ethics Committee of our hospital approved this retrospective study. As a retrospective study, informed consent was not required.

### Study design

**FIGURE 1:**
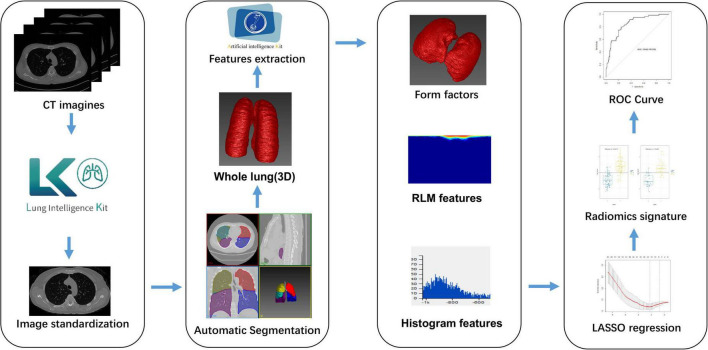
Flowchart showing how the radiomic signature was developed. CT, computed tomography; LASSO, least absolute shrinkage and selection operator; RLM, run length matrix; ROC, receiver-operator characteristic.

The study’s overall workflow comprised patient and image collection, region of interest (ROI) definition, radiomic feature extraction, modeling, and evaluating of performance. Figure 1 provides a brief overview of the modeling strategy.

### Patient selection

**FIGURE 2:**
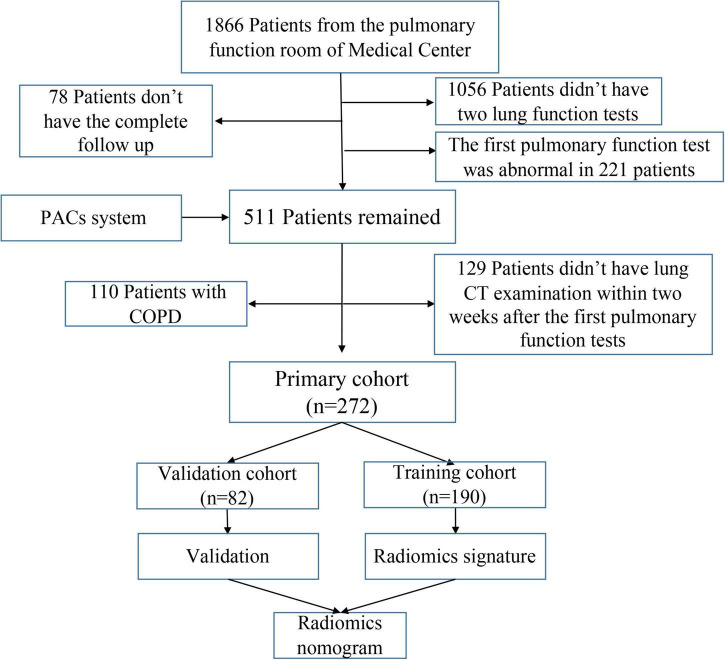
Flowchart for recruitment of patients in this study (PACS, picture archiving and communication system; COPD, chronic obstructive pulmonary disease; CT, computed tomography).

Patients with fSAD were diagnosed *via* pulmonary function tests (PFTs) in from July 2017 to August 2020 and collected retrospectively. The PFT results and chest CT images of the patients from a previous visit (with an interval of more than 1 year) were reviewed retrospectively. If the previous lung function results showed no fSAD, they were recruited into the study. The exclusion criteria were: (1) Patients who had not received two lung function tests; (2) lack of complete clinical data; (3) the interval between chest CT examination and the first pulmonary function examination was more than 2 weeks; and (4) patients with obstructive ventilation dysfunction. Finally, the inclusion criteria were satisfied by 272 patients who were thus recruited into this study. Figure 2 shows the flowchart for recruitment of patients in this study. Radiomic features were extracted from the patient’s chest CT scan. None of the patients had a sign of respiratory tract infection, wheezing in the lungs and a history of lung surgery. We extracted clinical parameters such as sex, age, symptoms, lung function index, smoking, asthma, and body mass index (BMI) from electronic medical record system, lung function room data, and telephone follow-up.

### Lung computed tomography protocol and pulmonary function tests

All the imaging data were from the picture archiving and communications system (PACS) of our medical center. The chest CT images were acquired on the same 64-slice multidetector CT scanners (Somatom Definition AS, Siemens). The CT protocol settings are shown in Supplementary material. The American Thoracic Society guidelines were followed when conducting the PFTs (diffusing capacity, lung volume, and spirometry) on a flow spirometer (Vmax22; SensorMedics), using as a reference the European Community Lung Health Survey values. At 15 h post-inhalation of albuterol (400 μg), post bronchodilation measurements were made. The ATS/ERS (American Thoracic Society/European Respiratory Society) recommendations were followed when carrying out forced expiratory flow (FEF) 25–75%, FEF50%, and FEF75%; forced vital capacity (FVC); forced expiratory volume in 1 s (FEV1); the ratio of FEV1 to FVC, maximal mid-expiratory flow (MMEF), maximum voluntary ventilation (MVV), vital capacity (VC), pre- and post-bronchodilator spirometry, and peak flow measurement (PEF).

To eliminate the influence of sex, age, height, weight, and other factors on the pulmonary function value, the pulmonary function index was presented as the percentage of the measured value compared with the predicted value (%PRED). Based on previous studies’ the recommendations and guidelines, the patients were classified into fSAD and non-fSAD (non-fSAD) groups. The fSAD diagnostic criteria were based on two or more of the following lung function indicators being less than 65% of the predicted value: forced expiratory flow 50%, forced expiratory flow 75%, and maximal mid-expiratory flow (6).

### Segmentation of the region-of-interest for image processing and extraction of radiomic features

The whole lung image was segmented automatically using Lung Kit (LK Version V1.0.0.R, GE Healthcare) from the baseline CT images, with a lung window width of 1,500 Hounsfield units (HU) and a window level of −400 HU [the other lung tissues (bronchus and vessels) were removed from the image]. Image standardization was carried out by preprocessing before auto-segmentation: (1) Image resampling: the resolution was resampled to a voxel size of 1.5 × 1.5 × 1.5 mm^3^; (2) intensity standardization: we re-ranged the gray value to 0–255. Two experienced respirologists, who were blinded to the clinical data, conducted manual modification of the CT images independently. Artificial Intelligence Kit Version 3.0.1.A (GE Healthcare) was used to extract the texture features from the segmented CT images (see Supplementary material I for details). Using an in-house software written in Python (Pyradiomics version 2.12),^1^ we extracted 326 radiomic features from each ROI for each patient. The extracted radiomic features included: Form factor parameter, gray level co-occurrence matrix (GLCM), run length matrix (RLM), texture parameters, and histograms. Supplementary material II shows the detailed description of the feature extraction. Patients in the cohort were divided randomly at 7:3 ratio into a training set (*n* = 190) and a validation set (*n* = 82). LASSO (least absolute shrinkage and selection operator) regression was then used to select the most useful predictive radiomic features from the training set (Supplementary Figure 1). Then, to predict fSAD for each patient, a radiomic signature was created as a linear depiction of the selected features, weighted by their respective coefficients. The accuracy of the predictions of the radiomic signature in the training and testing groups were evaluated using the area under the receiver-operator characteristic (ROC) curve (AUC).

### Construction and evaluation of a radiomics signature

The LASSO coefficients of the selected features were used to weight them to generate a radiomic signature. For each patient, we calculated the radiomic score (rad-score), and then used a linear kernel support vector machine (SVM) to construct the radiomic features according to the subset of top features from classified patients with fSAD to predict disease progression. Figure 1 shows the main process of building the radiomic signature. The details of SVM construction are shown in Supplementary material IV. The accuracy and stability of the SVM were assessed in comparison with alternative machine learning methods, such as Logistic regression, K-Near Neighbor (KNN), Bayes, Random Forest, and Decision Tree classifier. fSAD and non-fSAD subjects could then be grouped according to the predictions established using these methods models.

### Construction of the radiomic nomogram

Potential predictors were identified initially using univariate logistic regression. We then selected independent predictors of fSAD from among the potential predictive variables (sex, age, BMI, smoking history, asthma, tuberculosis, and bronchitis) using multivariate logistic regression. Finally, we used stepwise logistic regression to construct a joint prediction model from the independent predictors in the training set, and the performance of the joint model was verified using the data from the test sett. Then, we visualized the results using ROC curves and quantified the prediction performance using the AUC values. The good-ness-of-fit of this model was analyzed using the Hosmer-Lemeshow test. We assessed the agreement between the predicted disease progression probability and actual disease progression probability using Calibration curves. The clinical performance of the iconography in the two cohorts (training group and validation group) was assessed using Decision curve analysis (DCA).

### Statistical analysis

The mean and standard deviation (*SD*) were used to express continuous variables, and frequency and proportion were used to express categorical variables. Categorical variables were analyzed by a chi-squared or Fisher’s exact test. Continuous variables were assessed using a Mann–Whitney *U*-test or an independent sample *t*-test. Medcalc software (Version 15.2.2),^2^ R software (Version 3.5.1), Python (Version 2.12), and SPSS (Version 18.0; IBM) were employed to carry out the statistical analyses. Statistical significance was accepted at *p* < 0.05.

## Results

### People

The 272 recruited patients were divided randomly into the training group (n = 190) and the validation group (*n* = 82). The characteristics of the patients are shown in Table 1 and Supplementary Table 1. The rate of fSAD was not significantly different between the training and validation groups (59/190, 31.1% vs. 24/82, 29.3%, *P* = 0.769). No differences were observed among the other clinical parameters (sex, BMI, asthma, tuberculosis and bronchitis). However, in both the training and validation sets, age, asthma, and smoking differed significantly between the patients with fSAD and the non-fSAD subjects (Table 1).

**TABLE 1:** Preoperative clinical characteristics of patients with or without fSAD.

Characteristics	Training cohort (*n* = 190)		*P*-value	Validation cohort (*n* = 82)		*P*-value
	fSAD (*n* = 59)	Non-fSAD (*n* = 131)		fSAD (*n* = 24)	Non-fSAD (*n* = 58)	
Age, years			<0.001			<0.001
<60	7	56		3	43	
≥60	52	75		21	15	
Sex			0.779			0.776
Man	43	98		20	45	
Women	16	33		4	13	
BMI			0.023			0.018
<24	46	80		18	27	
≥24	13	51		6	31	
Smoking			<0.001			0.001
Yes	34	40		16	15	
No	25	91		8	43	
Asthma			<0.001			0.001
Yes	35	35		13	11	
No	24	96		11	47	
Tuberculosis			0.074			0.695
Yes	7	5		3	4	
No	52	126		21	54	
Bronchitis			0.469			0.109
Yes	11	19		8	10	
No	48	112		16	48	

BMI, body mass index; fSAD, small airway dysfunction.

### Feature selection

From each patient’s CT image, we extracted 328 imaging features. Initially, the Mann–Whitney test and ANOVA test identified 113 meaningful features. Sixty-four features remained after removing redundant features through Spearman correlation analysis. Finally, LASSO regression selected 17 predictive features, which were employed to construct the radiomic signature. Supplementary Table 2 and Supplementary Figure 2 show the feature details and the formula used to construct the radiomic signature.

### Radiomic signature’s performance and stability

**FIGURE 3:**
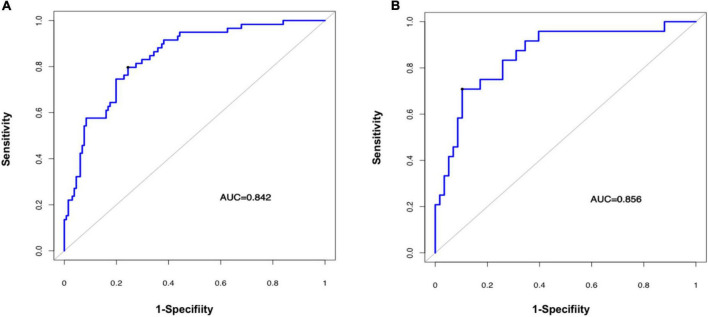
Evaluating the accuracy of the radiomic signature. Prediction of a high risk of fSAD using the radiomic signature in the training cohort **(A)** (AUC = 0.842) and in the validation cohort **(B)** (AUC = 0.856). AUC, area under the receiver-operator characteristic (ROC) curve; fSAD, small airway dysfunction.

**FIGURE 4:**
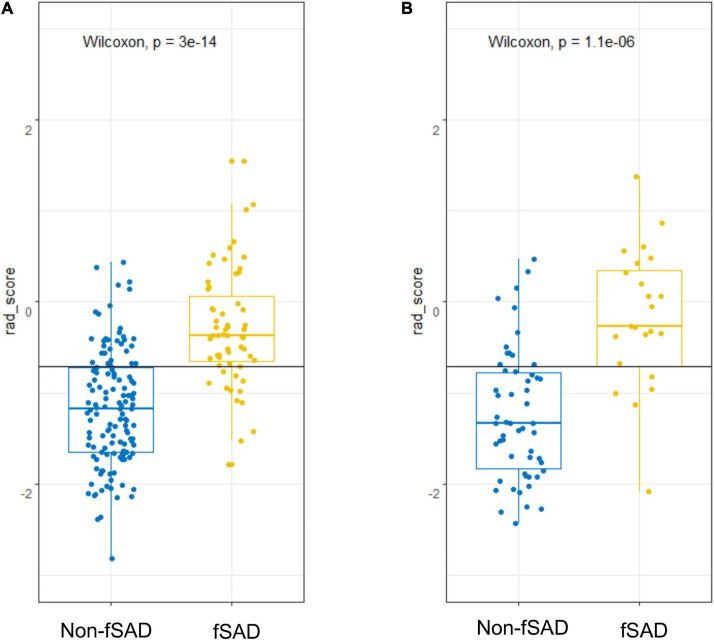
fSAD risk prediction using rad-score. Score dot diagrams showing the rad-score in **(A)** the training cohort and **(B)** the validation cohort. High fSAD risk is shown in yellow risk of fSAD, and low risk of fSAD is shown in blue. A high change of fSAD is indicated by a higher score. fSAD, small airway dysfunction; Rad-score, radiomic score.

The predictive performance of the rad-score in the two sets was evaluated using a ROC curve (Figures 3A,B). In general, patients with fSAD had higher rad-score values (training group: −0.298 vs. −1.168, *P* < 0.001, validation group: 0.188 vs. −1.335, *P* < 0.001). For the training set, the AUC value was 0.842 (95% CI: 0.782–0.891), with a sensitivity of 0.797 and specificity of 0.756. For the validation set, the AUC value was 0.856 (95% CI: 0.761–0.924), sensitivity was 0.708, and specificity was 0.897. Table 2 shows the predictive performance of the rad-score. Table 3 shows the comparison of the different model construction methods. Next we determined the quantitative scores of the radiomic signature for each patient with respect to the classification of risk assessment of fSAD, to show the effectiveness of the radiomic signature model at the individual level (Figures 4A,B).

**TABLE 2:** Predictive performance of the radiomic signature and radiomic nomogram.

Model	Radiomic signature			Radiomic nomogram		
	Specificity	Sensibility	AUC (95% CI)	Specificity	Sensibility	AUC (95% CI)
Training cohort	0.756	0.797	0.842 (0.782–0.891)	0.893	0.814	0.910 (0.860–0.946)
Validation cohort	0.708	0.897	0.856 (0.761–0.924)	0.879	0.875	0.933 (0.856–0.977)

AUC, area under the receiver operating characteristic (ROC) curve; CI, confidence interval.

**TABLE 3:** Discrimination performance of the different model construction methods in the fSAD and non-fSAD groups.

Method	Training cohort					Validation cohort				
	Accuracy	F1_ score	Sensitivity	Specificity	AUC	Accuracy	F1_ score	Sensitivity	Specificity	AUC
SVM	0.770	0.43	0.797	0.756	0.842	0.84	0.45	0.897	0.708	0.856
Logistic regression	0.674	0.061	0.982	0.301	0.667	0.78	0.130	0.996	0.203	0.640
Bayes	0.653	0.411	0.802	0.359	0.748	0.598	0.108	0.746	0.105	0.578
KNN	0.732	0.44	0.944	0.312	0.863	0.768	0.345	0.921	0.263	0.761
Decision Tree	0.837	0.627	0.993	0.4 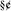	0.853	0.634	0.211	0.873	0.148	0.538
Random forest	0.784	0.453	0.985	0.304	0.922	0.671	0.229	0.927	0.148	0.630

AUC, area under the curve; CI, confidence interval; KNN, K-Near Neighbor; SVM, support vector machine.

### Construction of a radiomic nomogram

**FIGURE 5:**
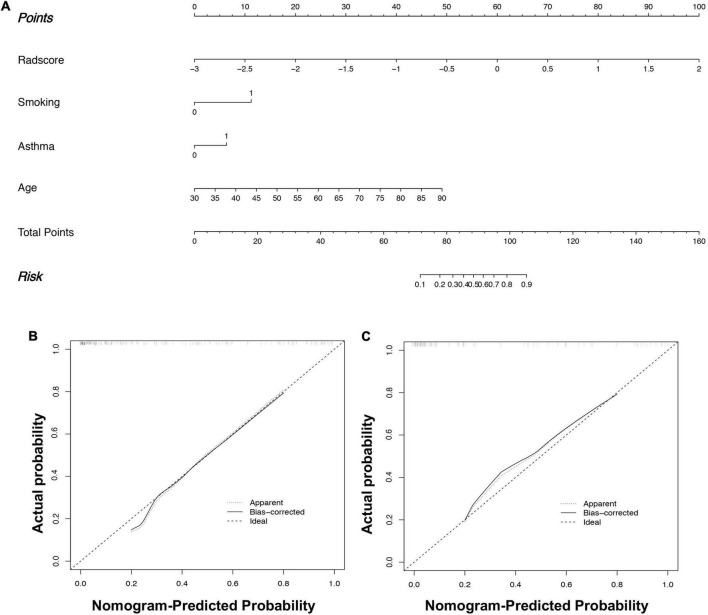
Radiomics nomogram **(A)** to predict fSAD. Using the training cohort data, the rad-score, age, smoking history, and asthma history were used to construct the radiomic nomogram. fSAD Calibration in **(B)** the training cohort and **(C)** the external validation cohort. The dashed reference line indicates the optimal nomogram. A dotted line represents the performance of the radiomic nomogram to predict high risk, and the solid line shows the correction of the nomogram’s deviation. fSAD, small airway dysfunction.

In the training set, by univariate analysis, age [odds ratio (OR): 1.072, 95% CI: 1.038–1.107, *P* < 0.001], BMI index (OR: 0.443, 95% CI: 0.218–0.901, *P* = 0.024), smoking history (OR: 3.094, 95% CI: 1.637–5.846, *P* = 0.001), SVM-signature (OR: 9.159, 95% CI: 4.521–18.553, *P* < 0.001), tuberculosis (OR: 3.392, 95% CI: 1.030–11.176, *P* = 0.045), and asthma history (OR: 4.000, 95% CI: 2.093–7.645, *P* < 0.001) displayed significant differences between the fSAD and non-FSAD groups. Multivariate logistic regression analysis identified the following as independent risk factors for fSAD, SVM-signature (OR: 12.447, 95% CI: 5.110–30.320, *P* ≤ 0.001), smoking history (OR: 3.586, 95% CI: 1.388–9.260, *P* = 0.008), asthma (OR: 2.507, 95% CI: 1.021–6.155, *P* = 0.045), and age (OR: 1.111, 95% CI: 1.059–1.167, *P* < 0.001) (Table 4). Age, asthma, and smoking history were then incorporated into the radiomic nomogram. A weighted number of points was then assigned to each factor. For each patient, the total number of points was determined according to the nomogram, and a higher points total correlated with an increased estimated probability of fSAD (Figure 5A).

**TABLE 4:** Stepwise logistic regression analysis predicting small airway dysfunction.

Variable	Univariate logistic regression			Multivariate logistic regression		
	OR	95% CI	*P*-value	OR	95% CI	*P*-value
SVM-signature	9.159	4.521–18.553	<0.001*	12.447	5.110–30.320	<0.001*
Age	1.072	1.038–1.107	<0.001*	1.111	1.059–1.167	<0.001*
Sex	1.105	0.551–2.217	0.779			
BMI	0.443	0.218–0.901	0.024*	0.923	0.809–1.053	0.232
Smoking	3.094	1.637–5.846	0.001*	3.586	1.388–9.260	0.008*
Asthma	4.000	2.093–7.645	<0.001*	2.507	1.021–6.155	0.045*
Tuberculosis	3.392	1.030–11.176	0.045*	3.501	0.704–17.415	0.126
bronchitis	1.351	0.597–3.055	0.470			

BMI, body mass index; CI, confidence interval; OR, odds ratio; SVM, support vector machine.

*p < 0.05.

### Performance evaluation of the prediction model

**FIGURE 6:**
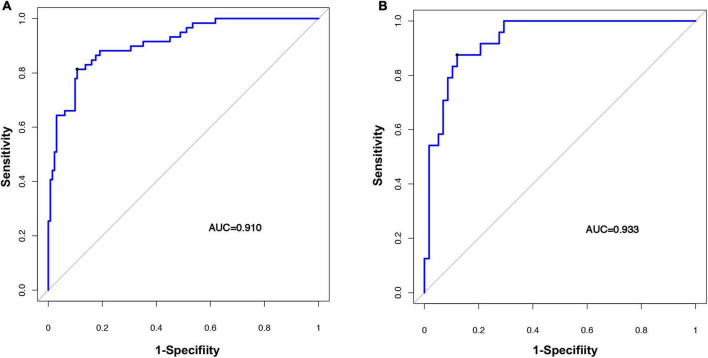
Evaluating the accuracy of the radiomic nomogram. The training cohort **(A)** and the validation cohort **(B)** were used to evaluate how accurately the radiomic nomogram predicted fSAD (AUC = 0.910 and 0.933, respectively). AUC, area under the receiver-operator characteristic (ROC) curve; fSAD, small airway dysfunction.

**FIGURE 7:**
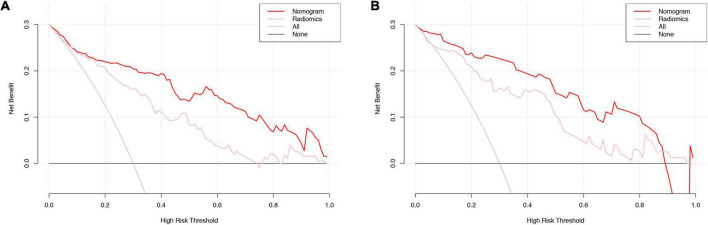
DCA curve evaluation of the clinical utility of the radiomic signature and the radiomic nomogram. Training cohort evaluation **(A)** and validation cohort evaluation **(B)**. The net benefit is shown on the y-axis. The threshold probability is shown on the x-axis. The maximum net benefit was obtained employing the radiomic nomogram (red line) in comparison with and the treat-none strategy (horizontal black line), the treat-all strategy (gray line), and the radiomic signature (pink line). DCA, decision curve analysis.

The radiomic nomogram was evaluated for its ability to judge and distinguish early fSAD. The discriminative ability of the developed nomogram was assessed using a ROC curve. The AUC value of training group was 0.910 (95% CI: 0.860–0.946), specificity: 0.893, and sensitivity: 0.814, The AUC value of validation group was 0.933, (95% CI = 0.856–0.977), specificity: 0.879, and sensitivity: 0.875 (Figures 6A,B and Table 2). Comparing the comprehensive prediction model and the actual observations of the two cohorts showed good consistency for predicted values using the calibration curve (Figures 5B,C). Finally, whether the nomogram could help with clinical prevention strategies was assessed using DCA curves. In the two cohorts, when the threshold probability changed between 0 and 1, the radiomic nomogram obtained the largest net benefit in comparison with the “all treatment” strategy, the “no treatment” strategy and a single radiomic signature (Figures 7A,B).

## Discussion

The present study investigated the prediction of fSAD in a Chinese population. The results showed that increasing age, cigarette smoking, asthma, and an SVM-signature were the main preventable risk factors. We developed a new imaging-based prediction model for the early identification of patients with fSAD. The texture features included high- and low-order radiomic features, similar to a previous study (12). We showed that LRHGLE emphasizes the combined measurement of image uniformity, allowing the quantification of the relationship between CT uniformity and attenuation, which was consistent with the research of Lafata et al’s. (12). And another previous study confirmed that the relationship between pulmonary function and imaging features (13). A high LRHGLE score reveals homogeneous and dense tissue, whereas a low LRHGLE score reveals heterogeneous tissue with low attenuation. The strong correlation coefficient of Short Run Low Gray Level Emphasis (SRLGLE) indicated that isolated intensity information is less important than spatially encoded texture information (13). However, linking a single image feature to the complex biological process of fSAD remains a challenge. Our developed radiomics score demonstrated a satisfactory prediction performance in the test and the validation groups.

In previous studies, fSAD was considered a precursor of COPD and asthma (10). However, it is difficult to detect early fSAD before the change of pulmonary function. We lack methods to determine which patients will develop fSAD that will eventually evolve into COPD (14). Currently, there are no effective predictive biomarkers for fSAD. In this study, a radiomics nomogram, including clinical factors (age, smoking etc.) and radiomic labels, was constructed, which showed a good predictive ability. In this study, we considered age as an independent predictor, which was supported by Miller’s findings that as people aged, their lung structure also aged, leading to a decline in lung function (15). Elderly people often show low lung oxygenation and reduced exercise ability and this age-related decrease in lung function correlates with lung structural remodeling (16). Studies have shown that lung parenchyma extracellular matrix structural remodeling causes an age-related decline in the elastic recoil capacity of the lung, which reduces the vital capacity, forced expiratory volume, and MMEF of the elderly, making them more prone to fSAD (15, 17, 18). Although age is closely related to fSAD, as a single factor, age is not sufficient to accurately predict the occurrence of fSAD.

Few fSAD-related risk factors have been identified. Herein, we identified that smoking increases the risk of fSAD. Some studies have shown that smoking can lead to a variety of severe airway dysfunctions in animals, and even in smokers without airway obstruction, quantitative CT measurement of emphysema can predict a significant decline in lung function (19). Our study confirmed that the risk of fSAD in smokers was significantly higher than that in non-smoking patients (20–24) and there is a close association between fSAD and smoking, supports efforts to strengthen smoking control in China to improve lung health.

There is controversy surrounding the link between fSAD and asthma (25). Herein, we found that asthma is an important predictor of fSAD. A previous study showed that patients with asthma will have more serious obstruction on PFTs, and with increasing age, allergic sensitization and multiple allergen sensitization gradually increase (26). However, in this study, a single history of asthma was not sufficient to explain the occurrence of fSAD in the later stage, which is related to the patient’s age and exposure to allergens. The association of fSAD with COPD requires further study. In addition, we lack evidence that lung structural injury can be prevented by fSAD prevention, diagnosis and treatment, as determined by spirometry (27).

Previous studies showed that BMI is associated with fSAD, and considered obesity as an fSAD risk factor (28). Obesity at any age can inhibit pulmonary function, and some weight loss studies indicated that in obese patients who lost weight, pulmonary function and symptoms improved (29, 30). However, because of our limited sample size, we could not confirm those results, which might be associated with racial and demographic factors.

Compared with PFTs, lung CT combined with radiomics shows the whole lung structure and reflects changes in pulmonary function, which is more comprehensive and timesaving. In addition, lung CT data is easily obtained, costs less, and shows a good predictive ability.

However, there are several limitations associated with the present study. Firstly, the retrospective nature of the study might have led to selection bias. Secondly, our sample size was relatively small. Third, the diagnosis of fSAD in this study was entirely based on PFTs, which are not as accurate as pathological examination. Therefore, our results apply only to fSAD defined by PFTs. In addition, although the selected time cut-off point was consistent with previous studies, further supporting evidence is required. Fourthly, environmental pollution exposure was an uncontrollable factor in our research. Genetic factors, bronchopulmonary dysplasia, bronchiectasis, premature delivery, birth weight, or birth age were not assessed in this study, which will be included in future research.

## Conclusion

In conclusion, we established a new prediction model that can effectively predict the early risk of fSAD, for which the major preventable risk is smoking. Thus, our findings reinforce comprehensive measures to control tobacco use and lung CT screening for early lung care. Accurately determining the possibility of fSAD would help to prevent early lung structural injury. Although our results are encouraging, further verification in larger and more diverse populations is required.

## Data availability statement

The data sets used and/or analyzed during this current study are available from the corresponding author upon reasonable request.

## Ethics statement

The studies involving human participants were reviewed and approved by the Ethics Committee of the Zhejiang Provincial People’s Hospital. The patients/participants provided their written informed consent to participate in this study. Written informed consent was obtained from the individual(s) for the publication of any potentially identifiable images or data included in this article.

## Author contributions

SC: manuscript draft, conception and design, study selection, data extraction, and review of final draft. ZS: conception and design, study selection, data extraction, provide technical support, and review of final draft. YM: collection and assembly of data and study selection. YL: collection and assembly of data, study selection, and statistical analysis. HW: collection and assembly of data and data analysis and interpretation. HC: data analysis and interpretation. JL: conception and design of the study and review of final draft. XG: conception and design and review of final draft. All authors have read and approved the final manuscript.

## Footnotes

1
https://pyradiomics.readthedocs.io/en/2.1.2/

2
https://www.MedCalc.org/

## References

[1] Bommart S Marin G Bourdin A Revel MP Klein F Hayot M Computed tomography quantification of airway remodelling in normal ageing subjects: A cross-sectional study. *Eur Respir J.* (2015) 45:1167–70. 10.1183/09031936.00215314 25537558

[2] Alfieri V Aiello M Pisi R Tzani P Mariani E Marangio E Small airway dysfunction is associated to excessive bronchoconstriction in asthmatic patients. *Respir Res.* (2014) 15:86. 10.1186/s12931-014-0086-1 25158694 PMC4243812

[3] Macklem PT. The physiology of small airways. *Am J Respir Crit Care Med.* (1998) 157:S181–3. 10.1164/ajrccm.157.5.rsaa-2 9606316

[4] Lee JE Choe KW Lee SW. Clinical and radiological characteristics of 2009 H1N1 influenza associated pneumonia in young male adults. *Yonsei Med J.* (2013) 54:927–34. 10.3349/ymj.2013.54.4.927 23709428 PMC3663245

[5] Izquierdo-Alonso JL Rodriguez-Gonzalezmoro JM de Lucas-Ramos P Unzueta I Ribera X Anton E Prevalence and characteristics of three clinical phenotypes of chronic obstructive pulmonary disease (COPD). *Respir Med.* (2013) 107:724–31. 10.1016/j.rmed.2013.01.001 23419828

[6] Xiao D Chen Z Wu S Huang K Xu J Yang L Prevalence and risk factors of small airway dysfunction, and association with smoking, in China: Findings from a national cross-sectional study. *Lancet Respir Med.* (2020) 8: 1081–93. 32598906 10.1016/S2213-2600(20)30155-7

[7] Konstantinos Katsoulis K Kostikas K Kontakiotis T. Techniques for assessing small airways function: Possible applications in asthma and COPD. *Respir Med.* (2016) 119:e2–9. 10.1016/j.rmed.2013.05.003 23764129

[8] van den Berge M Ten Hacken NHT Cohen J Douma WR Postma DS. Small airway disease in asthma and COPD: Clinical implications. *Chest.* (2011) 139:412–23. 10.1378/chest.10-1210 21285055

[9] Usmani OS Singh D Spinola M Bizzi A Barnes PJ. The prevalence of small airways disease in adult asthma: A systematic literature review. *Respir Med.* (2016) 116:19–27. 10.1016/j.rmed.2016.05.006 27296816

[10] Skylogianni E Triga M Douros K Bolis K Priftis KN Fouzas S Small-airway dysfunction precedes the development of asthma in children with allergic rhinitis. *Allergol Immunopathol.* (2018) 46:313–21. 10.1016/j.aller.2017.09.025 29338960

[11] Karimi R Tornling G Forsslund H Mikko M Wheelock A Nyren S Lung density on high resolution computer tomography (HRCT) reflects degree of inflammation in smokers. *Respir Res.* (2014) 15:23. 10.1186/1465-9921-15-23 24564813 PMC3944780

[12] Lafata KJ Zhou Z Liu JG Hong J Kelsey CR Yin FF. An exploratory radiomics approach to quantifying pulmonary function in CT images. *Sci Rep.* (2019) 9:11509. 10.1038/s41598-019-48023-5 31395937 PMC6687824

[13] Occhipinti M Paoletti M Bartholmai BJ Rajagopalan S Karwoski RA Nardi C Spirometric assessment of emphysema presence and severity as measured by quantitative CT and CT-based radiomics in COPD. *Respir Res.* (2019) 20:101. 10.1186/s12931-019-1049-3 31122243 PMC6533715

[14] Cho YH Seo JB Lee SM Kim N Yun J Hwang JE Radiomics approach for survival prediction in chronic obstructive pulmonary disease. *Eur Radiol.* (2021) 31:7316–24. 10.1007/s00330-021-07747-7 33847809

[15] Miller MR. Structural and physiological age-associated changes in aging lungs. *Semin Respir Crit Care Med.* (2010) 31:521–7. 10.1055/s-0030-1265893 20941653

[16] Piorunek T Kostrzewska M Stelmach-Mardas M Mardas M Michalak S Gozdzik-Spychalska J Small airway obstruction in chronic obstructive pulmonary disease: Potential parameters for early detection. *Adv Exp Med Biol.* (2017) 980:75–82. 10.1007/5584_2016_208 28197800

[17] Janssens JP Pache JC Nicod LP. Physiological changes in respiratory function associated with ageing. *Eur Respir J.* (1999) 13:197–205. 10836348 10.1034/j.1399-3003.1999.13a36.x

[18] Huang K Rabold R Schofield B Mitzner W Tankersley CG. Age-dependent changes of airway and lung parenchyma in C57BL/6J mice. *J Appl Physiol.* (1985) 2007:200–6. 10.1152/japplphysiol.00400.2006 16946023

[19] Yuan R Hogg JC Pare PD Sin DD Wong JC Nakano Y Prediction of the rate of decline in FEV(1) in smokers using quantitative Computed Tomography. *Thorax.* (2009) 64:944–9. 10.1136/thx.2008.112433 19734130 PMC3035577

[20] Wang C Xu J Yang L Xu Y Zhang X Bai C Prevalence and risk factors of chronic obstructive pulmonary disease in China (the China pulmonary health [CPH] study): A national cross-sectional study. *Lancet.* (2018) 391:1706–17. 10.1016/S0140-6736(18)30841-9 29650248

[21] Llontop C Garcia-Quero C Castro A Dalmau R Casitas R Galera R Small airway dysfunction in smokers with stable ischemic heart disease. *PLoS One.* (2017) 12:e0182858. 10.1371/journal.pone.0182858 28846677 PMC5573211

[22] Huang K Yang T Xu J Yang L Zhao J Zhang X Prevalence, risk factors, and management of asthma in China: A national cross-sectional study. *Lancet.* (2019) 394:407–18. 10.1016/S0140-6736(19)31147-X 31230828

[23] Wang M Luo X Xu S Liu W Ding F Zhang X Trends in smoking prevalence and implication for chronic diseases in China: Serial national cross-sectional surveys from 2003 to 2013. *Lancet Respir Med.* (2019) 7:35–45. 10.1016/S2213-2600(18)30432-6 30482646

[24] Collaborators GBDT. Smoking prevalence and attributable disease burden in 195 countries and territories, 1990-2015: A systematic analysis from the global burden of disease study 2015. *Lancet.* (2017) 389:1885–906. 10.1016/S0140-6736(17)30819-X 28390697 PMC5439023

[25] Postma DS Brightling C Baldi S Van den Berge M Fabbri LM Gagnatelli A Exploring the relevance and extent of small airways dysfunction in asthma (ATLANTIS): Baseline data from a prospective cohort study. *Lancet Respir Med.* (2019) 7:402–16. 10.1016/S2213-2600(19)30049-9 30876830

[26] Nagarajan S Ahmad S Quinn M Agrawal S Manilich E Concepcion E Allergic sensitization and clinical outcomes in urban children with asthma, 2013-2016. *Allergy Asthma Proc.* (2018) 39:281–8. 10.2500/aap.2018.39.4147 30095393 PMC6052173

[27] Chen H Zeng QS Zhang M Chen RC Xia TT Wang W Quantitative low-dose computed tomography of the lung parenchyma and airways for the differentiation between chronic obstructive pulmonary disease and asthma patients. *Respiration.* (2017) 94:366–74. 10.1159/000478531 28738344

[28] Littleton SW Tulaimat A. The effects of obesity on lung volumes and oxygenation. *Respir Med.* (2017) 124:15–20. 10.1016/j.rmed.2017.01.004 28284316

[29] Juel CT Ali Z Nilas L Ulrik CS. Asthma and obesity: Does weight loss improve asthma control? A systematic review. *J Asthma Allergy.* (2012) 5:21–6. 10.2147/JAA.S32232 22791994 PMC3392696

[30] Eneli IU Skybo T Camargo CA Jr. Weight loss and asthma: A systematic review. *Thorax.* (2008) 63:671–6. 10.1136/thx.2007.086470 18663068

