# Supplementary material for: A novel computed tomography radiomic nomogram for early evaluation of small airway dysfunction development
Source: Front Med (Lausanne). 2022 Sep 13;9:944294. doi: 10.3389/fmed.2022.944294 (PMC9513435; doi:10.3389/fmed.2022.944294)
Supplement: Supplementary file 1 [file Data_Sheet_1.docx]

**Supplementary material**

**I. Computed tomography (CT) image acquisition**

The conditions for lung CT examination were as follows: 120 kV, 10–40 mA, gantry rotation speed (0.5 seconds), and helical scan mode (pitch 1.2). All lung CT images were reconstructed at a slice thickness of 2 mm, with a spacing interval of 2 mm. All images were obtained at a lung window width of 1,500 Hounsfield units (HU) and a window level of −400 HU.

**II. Radiomic Features Extraction**

**2.1 Dimension reduction of radiomic features**

First, the extracted texture features were standardized (z-score), which removed the unit limits of the data of each feature such that the indexes of different units or orders could be compared and weighted. Then, z-score normalization was used to make the image intensities have the properties of a standard normal distribution, with $\mu=1$ and $\sigma=0$, where $\mu$ is the mean value of the images, and is the standard deviation. The normalized values (also called z scores) of the image intensities (x) were calculated as follows:

$$z=\frac{\chi-\mu}{\sigma}$$

Then, feature dimensionality reduction was performed as follows. Analysis of variance (ANOVA) and the Mann–Whitney U test (MW) dimensionality reduction were performed, and then the correlation value was calculated to reduce data redundancy. The software calculated the paired correlation between each pair of features. If the Spearman correlation coefficient was greater than 0.9, it was believed to approach a linear relationship. In other words, the two features could express each other, which had no effect on the result; however, the coefficient was different when fitting. In this study, the software automatically removed the feature of the latter when the two features were comparatively relevant. All the above steps were carried out using the AK software. Then, the least absolute shrinkage and selection operator (LASSO) was used to select the most useful predictive radiomic features.

**2.2 The LASSO algorithm**

LASSO is a powerful algorithm for regression analysis with high dimensional predictors. In the present study, the LASSO algorithm was combined with a logistic regression model for model development. We used the LASSO logistic regression model to select the most important predictive features and to construct a radiomic signature in the training set. This algorithm minimizes a log partial likelihood subject to the sum of the absolute values of the parameters bounded by a constant:

$\hat{\beta}=arg\min\mathcal{l}\left( \beta\right)$ subject to $\sum\left| \beta_{i} \right|\leq t$, where $\hat{\beta}$ is the obtained parameter, $\mathcal{l}\left( \beta\right)$ is the log partial likelihood of the logistic regression model, and $t>0$is a constant.

The LASSO algorithm shrinks some coefficients and reduces others to exactly 0 via the absolute constraint. Thus, LASSO is an outstanding method for feature selection that retains the good features of both subset selection and ridge regression. In this study, the constant t was set as 0.015, and LASSO selected nonzero coefficients, `1$\hat{\beta}$, and a formula was generated using a linear combination of the selected features that were weighted by their respective LASSO coefficients. The “glmnet” package in the R statistical software version 3.4.1 was used for LASSO logistic regression model analysis; the specific formula is as follows.

Radiomics signature calculation formula:

Rad-score = −0.422 * Percentile70 + 0.069 * ClusterShade_AllDirection_offset1_SD

+ 0.276 * Correlation_angle45_offset9 + 0.358 * Inertia_AllDirection_offset2_SD + −0.088 * InverseDifferenceMoment_AllDirection_offset2_SD + 0.38 * InverseDifferenceMoment_AllDirection_offset6_SD + −0.014 * GrayLevelNonuniformity_AllDirection_offset1_SD + 0.054 * HighGrayLevelRunEmphasis_AllDirection_offset8_SD + 0.175 * LongRunEmphasis_angle0_offset2 + 0.042 * LongRunEmphasis_angle0_offset7 + −0.057 * LowGrayLevelRunEmphasis_AllDirection_offset2_SD + −0.285 * RunLengthNonuniformity_AllDirection_offset1_SD + 0.082 * ShortRunEmphasis_AllDirection_offset3_SD + 0.014 * ShortRunEmphasis_AllDirection_offset8_SD + 0.1 * ShortRunEmphasis_angle0_offset3 + −0.055 * ShortRunLowGrayLevelEmphasis_AllDirection_offset4_SD + 0.014 * Compactness1

Supplementary Figure. 2 shows the flowchart of the radiomic features dimension reduction. Supplementary Table 2 shows the radiomic features classification and calculation formula of the texture features after dimension reduction.

**2.3 Detailed descriptions of the features**

**2.3.1 Information on the features**

Three hundred and twenty-eight radiomic features from each phase were calculated using the AK software. Radiomic features included Histogram, Formfactor, Gray-Level Co-occurrence Matrix (GLCM), and Run length matrix (RLM).

**2.4 Details of GLCM**

**Co-occurrence matrices**

In the GLCM, $P\left( i,\left. j \right|\theta,d \right)$ represents the joint probability of certain sets of pixels having certain gray-level values. It calculates how many times a pixel with gray-level $i$occurs jointly with another pixel having a gray value j, by varying the displacement vector$d$between each pair of pixels.

The advantage of the co-occurrence matrix calculations is that the co-occurring pairs of pixels can be spatially related in various orientations with reference to distance and angular spatial relationships, considering the relationship between two pixels at a time. As a result, the combination of gray levels and their positions are exhibited. Therefore, it is defined as “A two-dimensional histogram of gray levels for pair of pixels, which are separated by a fixed spatial relationship”. However, the matrix is sensitive to rotation. Changes in different offsets define pixel relationships by varying directions.

The rotation angle of an offset: 0°, 45°, 90°, 135°, and displacement vectors (distance to the neighbor pixel: 1, 2, 3 ...), result in different co-occurrence distributions from the same reference image. GLCM of an image is computed using displacement vector d defined by its radius, (distance or count to the next adjacent neighbor is preferably equal to one) and rotational angles.

**III. Definition of net benefit in the decision curve analysis.**

Decision curve analysis (DCA) was used to assess the clinical utility of the radiomic nomogram model in the three cohorts. The "true" positive and weighted false-positive rates were calculated across different threshold probabilities in the validation set to determine the net benefit. Specifically, the weighting factor was defined as the specific value of the threshold probability divided by 1 minus the threshold probability. A higher true-positive rate and a relatively low false-positive rate were suggested by a high net benefit. Plotting the net benefit against the threshold probability across the range of 0 to 0.8 generated the decision curve.

The net benefit was defined by the following equation:

Net Benefit = $TPR*\omega-\frac{P_{t}}{1-P_{t}}*FPR*\left( 1-\omega\right),$

where 𝑃𝑡 is the “threshold possibility” to stratify the patients into small airway dysfunction (fSAD) or non-fSAD groups. Patients with a probability of having fSAD higher than 𝑃𝑡 are high-risk patients. These patients would be recommended for aggressive intervention(s), while others (non-fSAD) do not need intervention. The true positive rate (TPR) is defined as the proportion of high-risk patients among the patients having fSAD. The false positive rate (FPR) is defined as the proportion of high-risk patients among the patients in the non-fSAD group. 𝜔, is the prevalence of having fSAD, calculated by dividing the total number of patients by the number of patients with fSAD. In the condition of “treat none”, no patient is classified as high risk, both the TPR and FPR are zero, thus the net benefit is zero. In the condition of “treat everyone”, all patients are classified as high risk (TPR = FPR = 1), thus the Net Benefit is calculated as

$${Net Benefit}_{Treat Everyones}=\omega-\frac{P_{t}}{1-P_{t}}*\left( 1-\omega\right)=\frac{1-\omega}{P_{t}-1}+1$$

, which is a monotonically decreasing curve in the figure.

IV. Support vector machine (SVM) construction

The relative standard deviation (RSD) and bootstrap method were used to quantify the stability of each machine learning method. For each classification method, we trained the model on the secondary sampling training queue (size n / 2) in the training set or the test set, and used the area under the receiver operating characteristic (ROC) curve (AUC) to evaluate its performance on the remaining data. The bootstrap method was used to conduct 100 secondary samplings on the training set or test set. The RSD is the absolute value of the coefficient of variation, usually expressed as a percentage according to the equation:

$$\mathrm{RSD}=\frac{\sigma_{\mathrm{AUC}}}{\mu_{\mathrm{AUC}}}\times100$$

where σAUC and µAUC are the standard deviation and mean of the 100 AUC values, respectively. It should be noted that higher stability corresponds to lower RSD values.

**References:**

[1] Kerr KF, Brown MD, Zhu K, Janes H. Assessing the Clinical Impact of Risk Prediction Models With Decision Curves: Guidance for Correct Interpretation and Appropriate Use. Journal of Clinical Oncology 2016; 34(21):2534-40 doi10.1200/JCO.2015.65.5654.

[2] Vickers AJ, Van Calster B, Steyerberg EW. Net benefit approaches to the evaluation of prediction models, molecular markers, and diagnostic tests. The BMJ 2016;352:i6. doi10.1136/ bmj.i6.

**Fig. S1 LASSO logistic regression to select radiomic features.**


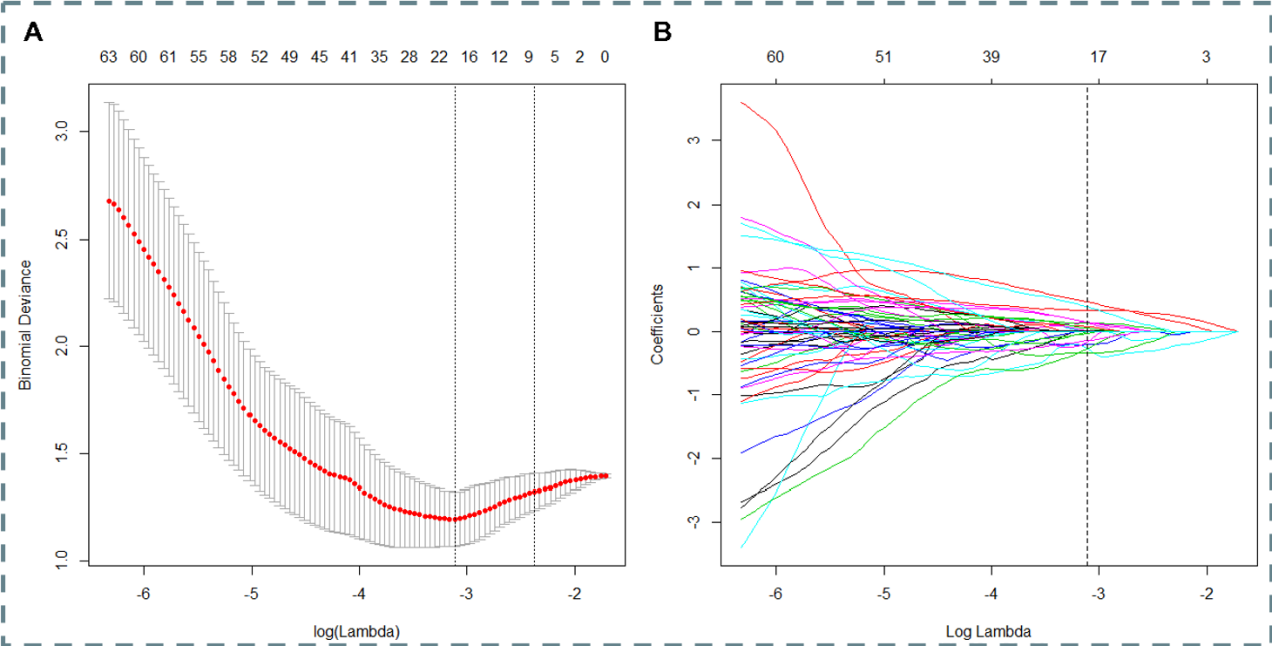


(a) In the LASSO model, for 10-fold cross-validation, the tuning parameter ( λ) was chosen as the minimum criterion. The x-axis shows the value of log (λ) and the y-axis shows the binominal deviance in the 10-fold cross-validation. The relationship between partial likelihood deviation and log λ is drawn. Dashed vertical lines at the best values were drawn using the minimum criteria and 1-SE criteria. (b) The 64 texture features identified in the LASSO coefficient profile. Ten-fold cross-validation was used to draw the vertical line in the log λ sequence at the selected value, which indicated 17 features with non-zero coefficients.

**Fig. S2 Flowchart of radiomic features dimension reduction.**

Supplementary Table 1 shows the characteristics of the study population

| Table S1. Characteristics of the study population | | | | | |
| --- | --- | --- | --- | --- | --- |
| Characteristics | Training cohort (n = 190) | | Validation cohort (n = 82) | | P value |
|  | n | % | n | % |  |
| Age, years |  | |  | | 0.091 |
| < 60 | 63 | 33.1 | 36 | 43.9 |  |
| ≥ 60 | 127 | 66.9 | 46 | 56.1 |  |
| Sex |  | |  | | 0.372 |
| Man | 141 | 74.2 | 65 | 79.3 |  |
| Women | 49 | 25.8 | 17 | 20.7 |  |
| BMI |  | |  | | 0.073 |
| < 24 | 126 | 66.3 | 45 | 54.9 |  |
| ≥ 24 | 64 | 33.7 | 37 | 45.1 |  |
| Smoking |  | |  | | 0.859 |
| Yes | 74 | 38.9 | 31 | 37.8 |  |
| No | 116 | 61.1 | 51 | 62.2 |  |
| Asthma |  | |  | | 0.180 |
| Yes | 70 | 36.8 | 24 | 29.3 |  |
| No | 120 | 63.2 | 58 | 70.7 |  |
| Tuberculosis |  | |  | | 0.510 |
| Yes | 12 | 6.3 | 7 | 8.5 |  |
| No | 178 | 93.7 | 75 | 91.5 |  |
| Bronchitis |  | |  | |  |
| Yes | 30 | 15.8 | 18 | 22.0 | 0.221 |
| No | 160 | 84.2 | 64 | 78.0 |  |
| Small airway dysfunction |  | |  | | 0.769 |
| Yes | 59 | 68.9 | 24 | 29.3 |  |
| No | 131 | 31.1 | 58 | 70.7 |  |
| BMI, body mass index | | | | | |

Table 2 shows the radiomic features classification and calculation formula of the texture features after dimension reduction.

| Table S2. The classification and calculation formula of the 10 texture features | | |
| --- | --- | --- |
| Histogram | Percentile70 | $n=\frac{P}{100}*N$ |
| Texture | ClusterShade_AllDirection_offset1_SD | $\sum_{i,j} \left( \left( i-u \right)+\left( j-u \right) \right)^{3}g\left( i,j \right)$ |
|  | Inertia_AllDirection_offset2_SD | 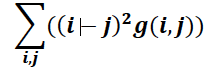 |
| GLCM | Correlation_angle45_offset9 | $\sum_{i,j} \frac{\left( i-u \right)\left( j-u \right)g\left( i,j \right)}{\sigma^{2}}$ |
|  | InverseDifferenceMoment_AllDirection_offset2_SD | $\sum\sum\frac{1}{1+{（i-j）}^{2}}g\left( i,j \right)$ |
|  | InverseDifferenceMoment_AllDirection_offset6_SD |  |
| RLM | GrayLevelNonuniformity_AllDirection_offset1_SD | $\mathrm{GLN}\left( \theta\right)=\frac{1}{n_{r}}\sum_{i=1}^{M} \left( \sum_{j=1}^{N} p\left( i,j,\theta\right) \right)^{2}$ |
|  | LongRunEmphasis_angle0_offset7 | $\mathrm{LRE}\left( \theta\right)=\frac{1}{n_{r}}\sum_{i=1}^{M} {\sum_{j=1}^{N} p\left( i,j,\theta\right)j}^{2}$ |
|  | LongRunEmphasis_angle0_offset2 |  |
|  | LowGrayLevelRunEmphasis_AllDirection_offset2_SD | $\mathrm{LGRE}\left( \theta\right)=\frac{1}{n_{r}}\sum_{j=i}^{N} {\sum_{i=1}^{M} \frac{p\left( i,j,\theta\right)}{i^{2}}}$ |
|  | HighGrayLevelRunEmphasis_AllDirection_offset8_SD | $\mathrm{HGRE}\left( \theta\right)=\frac{1}{n_{r}}\sum_{j=i}^{N} {\sum_{i=1}^{M} p\left( i,j,\theta\right)i}^{2}$ |
|  | RunLengthNonuniformity_AllDirection_offset1_SD | $\mathrm{RLN}\left( \theta\right)=\frac{1}{n_{r}}\sum_{j=i}^{N} ({\sum_{i=1}^{M} p\left( i,j,\theta\right))}^{2}$ |
|  | ShortRunEmphasis_AllDirection_offset3_SD | $\mathrm{SRE}\left( \theta\right)=\frac{1}{n_{r}}\sum_{i=1}^{M} {\sum_{j=1}^{N} \frac{p\left( i,j,\theta\right)}{i^{2}}}$ |
|  | ShortRunEmphasis_AllDirection_offset8_SD |  |
|  | ShortRunEmphasis_angle0_offset3 |  |
|  | ShortRunLowGrayLevelEmphasis_AllDirection_offset4_SD | $\mathrm{SRLGE}\left( \theta\right)=\frac{1}{n_{r}}\sum_{j=i}^{N} {\sum_{i=1}^{M} \frac{p\left( i,j,\theta\right)}{{i^{2}j}^{2}}}$ |
| Form Factor | Compactness1 | $compactness 1=\frac{V}{\sqrt{\pi}A^{\frac{2}{3}}}$ |
